# Uncovering the Advantages of Foam Dressings with Active Ingredients

**DOI:** 10.3390/ph18060768

**Published:** 2025-05-22

**Authors:** Daniela Chrysostomou, Georgios E. Papanikolaou, Lorraine Boshoff, Thandazi Mbele, Andrea Pokorná, Adéla Holubová, Frank A. D. T. G. Wagener, Niels A. J. Cremers

**Affiliations:** 1 Department of Health Sciences, Faculty of Medicine, Masaryk University, 625 00 Brno, Czech Republic; danielachrys@hotmail.com (D.C.); apokorna@med.muni.cz (A.P.); 2 Health@45, Johannesburg 1612, South Africa; lorraine.boshoff@gmail.com (L.B.); thandimbhele@gmail.com (T.M.); 3 GP Plastic Surgery Private Practice, 45444 Ioannina, Greece; info@gp-plasticsurgery.gr; 4 Department of Health Sciences, College of Polytechnics Jihlava, 586 01 Jihlava, Czech Republic; 5 Faculty of Health and Social Sciences, University of South Bohemia, 370 11 České Budějovice, Czech Republic; adela.holubova@diapodicare.cz; 6 DiaPodi Care, spol. s r.o., 392 01 Soběslav, Czech Republic; 7 Department of Dentistry-Orthodontics and Craniofacial Biology, Radboudumc, 6525 EX Nijmegen, The Netherlands; frank.wagener@radboudumc.nl; 8 Triticum Exploitatie BV, 6222 NK Maastricht, The Netherlands; 9 Department of Gynecology and Obstetrics, Maastricht University Medical Center, 6202 AZ Maastricht, The Netherlands

**Keywords:** foam dressings, active dressings, bandages, polyurethanes, wounds, wound healing, exudates, honey, silver, cost–benefit analysis

## Abstract

**Background/Objectives:** Foam dressings are designed for their ability to manage exudate and are selected to optimize wound repair. Various foam dressings are available, ranging from basic polyurethane to more sophisticated options, incorporating active components to combat infections or foster healing. This study investigates the requirements for the most suitable foam dressing through a combination of field research, laboratory testing, and clinical evaluation. **Methods**: We tested 17 foam dressings commonly used by wound care professionals while attending an international conference. An effective foam dressing should absorb wound fluid for several days, as wound care professionals value absorption and retention capacity, often favoring less frequent changing dressings, preferably twice a week or even weekly. **Results**: The foam dressings tested can absorb the expected amount of exudate typically produced by different wound types. There is some variability in retention capacity and product prices, resulting in differences in cost-effectiveness among products. In addition, some dressings are enriched with active ingredients that can accelerate healing through their antimicrobial and anti-inflammatory properties, such as foam dressings infused with silver or honey. A honey-based foam dressing was evaluated in a clinical survey involving eight wound care specialists, and four clinical cases with varying wound pathologies were discussed in more detail to highlight its key properties. **Conclusions**: Ideally, a foam dressing should have adequate absorption and retention capacities, effectively resolve and prevent infections, protect against external trauma, ensure optimal patient comfort without damaging newly formed granulation tissue, accelerate wound healing processes, and reduce wound care time (e.g., remaining in place for 7 days). Together, these factors make honey- or silver-loaded foam dressings more cost-effective than plain dressings due to their antimicrobial activities and ability to nourish tissues.

## 1. Introduction

Many intrinsic and extrinsic factors influence wound-healing processes [1]. Moist wounds heal faster than dry or wet wounds, according to a study conducted in 1962 by Winter et al. [2]. They demonstrated in a porcine model that the rate of epithelialization of moist wounds was twice as fast compared to dry conditions. In 1963, a study on humans confirmed that moist wounds heal faster than air-exposed dry wounds [3]. A simple explanation is that dry wounds promote the formation of crusts and scabs, hindering re-epithelialization by forming a physical barrier for migrating new epithelial cells [3,4]. In wet wounds, an excessive amount of wound fluid (exudate) on the wound bed leads to maceration, resulting in the softening and breakdown of skin. Moist wounds heal 2–3 times faster than dry wounds [5]. Although wound exudate is part of the natural healing process, a wrong composition (e.g., with high levels of proteases or inflammatory cytokines) or exudate excess can keep the wound healing process in a non-healing inflammatory state [6,7]. Clinical problems associated with excessive exudate include leakage and soiling of dressings, malodor, increased risk of infection, frequent dressing changes, higher costs, and discomfort or pain [8,9]. Therefore, it is essential to control the level of wound exudate. Different types of wound dressings are considered appropriate for heavily exuding wounds.

Many wound dressings are available on the market, varying in materials and formulations [7]. An ideal dressing should adhere to several properties, such as effective exudate handling capacity (including retention even under compression), ease of application, and removal with minimal pain and no side effects, all while optimizing patient comfort and movement. Additional properties, such as the incorporation of antiseptics, antimicrobials, or agents that can enhance healing (e.g., by facilitating autolytic debridement or regulating protease and inflammatory mediator levels), can be valuable depending on the wound type [10]. Together with wear time, the frequency of dressing changes, the need for secondary dressings, and pricing, these factors will define the cost-effectiveness of the product.

Wound care products that are more appropriate for managing various levels of exudate include cotton, polyester, viscose fibers or fabrics, and foam dressings [11,12]. They can absorb low, moderate, and high exudate levels, though their capacity to retain moisture, particularly under pressure, varies. Dressings made from cotton, viscose, or polyester textiles are mainly used as secondary dressings, while foam dressings can be utilized as either primary or secondary dressings and provide some cushioning. Gel-forming materials are mainly indicated as primary dressings and may help reduce lateral tracking of wound fluid and the risk of peri-wound maceration. However, depending on the material, they can handle different levels of exudate (for example, hydrocolloid dressings should be used for dry or low-exuding wounds and alginates are suitable for low to moderate levels of exudate, while carboxymethyl cellulose and super absorbent dressings are recommended for moderate to high exudate levels) [13].

Foam dressings have multiple characteristics and are designed to meet one of the primary objectives of wound care: creating a moist environment that promotes wound healing (Table 1). They are generally made from semi-permeable polyurethane, are non-adherent and non-linting, and allow water vapor to enter while blocking bacteria and other contaminants [13]. The outer layer may be waterproof or hydrophobic. Foam dressings are available in various sizes and shapes, and some come with adhesive tape or borders around the edges for easier application and better adhesion even in places that are more problematic for the application of dressing. Their versatility enables use as either primary or secondary dressings, particularly for wounds with moderate to high exudate levels. Foam dressings also support autolytic debridement, offer cushioning against mechanical stress (e.g., shear and pressure), and can be combined with active agents such as silver or honey to enhance antimicrobial or healing properties [10,13,14]. Depending on their composition and design, some foam dressings are better suited for cavity wounds or use under compression therapy, while other dressings improve application and patient comfort or provide extra wound healing benefits.

This study investigates the requirements for the most suitable foam dressing through a combination of field research, laboratory testing, and literature data. First, we conducted a field study among clinicians to investigate which foam dressings are most commonly used and to understand what they consider the most important aspects of foam dressings. The absorption and retention capacities of a range of commonly used plain (non-enriched) and active foam dressings were examined in a laboratory study. Subsequently, we evaluated how these properties relate to literature data on the amounts of exudate. To explore the potential additive value of honey in foam dressings, we surveyed wound care clinicians using a medical-grade honey-based foam dressing and collected case materials to demonstrate specific characteristics.

## 2. Results

### 2.1. Outcome Questionnaire Among Wound Care Conference Attendees

Thirty-nine wound care specialists filled in a short questionnaire with questions related to foam dressings. Most of them were nurses (*n* = 32, 82.1%), followed by physicians (*n* = 5, 12.8%), and industry representatives (*n* = 2, 5.1%). Foam dressings identified as frequently used by specialists included Mepilex (66.7%), Allevyn non-adhesive (51.3%), Aquacel Ag Foam (46.2%), Biatain non-adhesive (46.2%), Mepilex Ag (38.5%), Allevyn Ag non-adhesive (35.9%), Tegaderm Foam (25.6%), Suprasorb foam (23.1%), Cutimed Siltec (15.4%), and Polymem Max (15.4%). Other dressings were used by less than 10% of the wound care specialists. The average frequency of dressing changes by these wound care specialists was daily (12.8%), every other day (23.1%), twice a week (43.6%), and once a week (20.5%). The most important aspects of a foam dressing according to these wound care specialists were: absorption of exudate (79.5%), comfort for patients (69.2%), frequency of dressing change (41%), ease in use (application/removal) (41%), and retention of exudate (38.5%).

### 2.2. Prices per Foam Dressing

**Figure 1:**
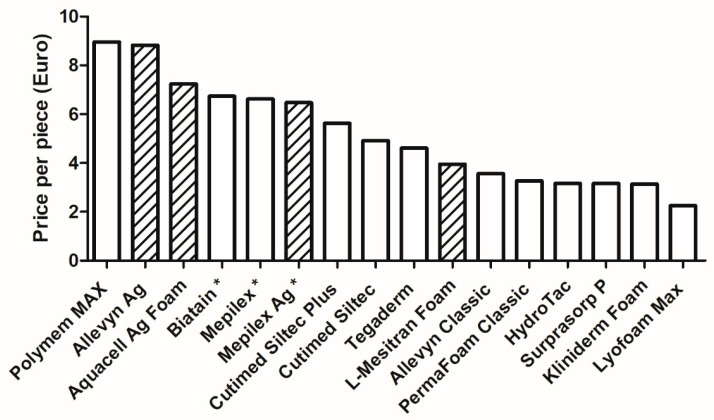
Overview of the prices per single foam dressing. Most dressings were 10 cm by 10 cm, except those with an * (Polymem Max 11 cm × 11 cm, Biatain 10 cm × 20 cm, Mepilex 10 cm × 20 cm, and Mepilex Ag 10 cm × 21 cm). The columns with diagonal lines are “active” foam dressings.

All foam dressings for the tests were ordered online, except L-Mesitran Foam, which is produced in-house. We included the dressings based on the survey results and their availability for shipping to our office in the Netherlands and ordered the product from the online shop at the lowest price. Also, we included the online price of L-Mesitran Foam. An overview of the prices per dressing is calculated (depending on the pieces per box) and presented in Figure 1. For most dressings, the size was 10 by 10 cm, and the quantity per box was 10 pieces; see Table 2. Four products had other dimensions, i.e., Polymem Max 11 cm × 11 cm, Biatain 10 cm × 20 cm, Mepilex 10 cm × 20 cm, and Mepilex Ag 10 cm × 21 cm. The latter three products came in boxes with 5 pieces instead of 10 pieces. There is quite a difference between the lowest and highest prices per dressing (EUR 2.25 for Lyofoam Max versus EUR 8.96 for Polymem Max). The active foam dressings (enriched with other ingredients) are among those with the highest price (between EUR 6.47 and EUR 8.83), except L-Mesitran Foam (EUR 3.95). The prices of the remaining dressings range from EUR 3.14 (Kliniderm Foam) to EUR 5.63 (Cutimed Siltec Plus). Since not all products come in dimensions of 10 cm by 10 cm (100 cm^2^), this may impact the price per dressing for the dressings that come in different sizes. To account for the different sizes, the prices per 100 cm^2^ were calculated. However, please note that dressings in larger sizes are often more cost-effective to purchase than smaller pieces; therefore, this modulation may not be optimal as well.

### 2.3. Thickness and Weight of the Dressings

**Figure 2:**
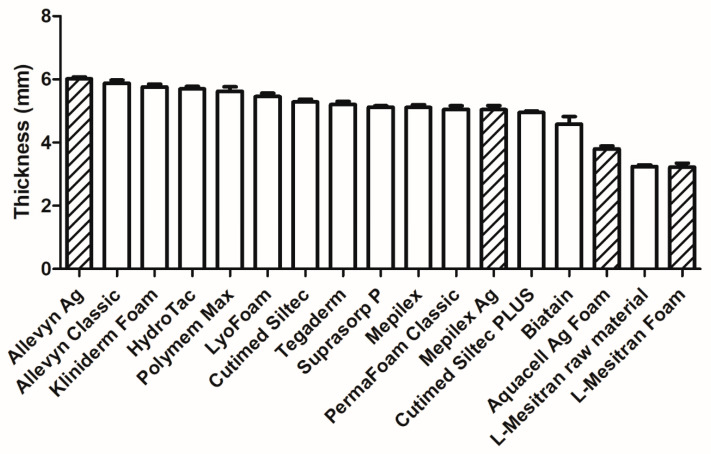
Thickness of different foam dressings in mm. Twenty individual dressings were measured per brand, and mean +/− SD is presented. The columns with diagonal lines are “active” foam dressings.

The length and width of the included dressings were measured. The maximum dimensions of each dressing fit well within the specifications, allowing for up to 2 mm variation on both sides (e.g., 9.8 cm by 10.2 cm when the dressing should be 10 cm by 10 cm). For most dressings, there was a rounded corner, except for Polymem Max and L-Mesitran Foam. This was likely the result of the manufacturing method: cutting or punching, with the punched dressings having a rounded corner. Mepilex, Mepilex Ag, Cutimed Siltec, and Cutimed Siltec Plus were not rectangular and differed in shape. The thickness of all dressings was carefully measured, see Figure 2. The thickness of the dressings varied between 3.2 and 6.0 mm, with most dressings being 5 or 6 mm. L-Mesitran Foam had the lowest thickness, followed by Aquacell Ag Foam (3.8 mm) and Biatain non-adhesive (4.6 mm).

**Figure 3:**
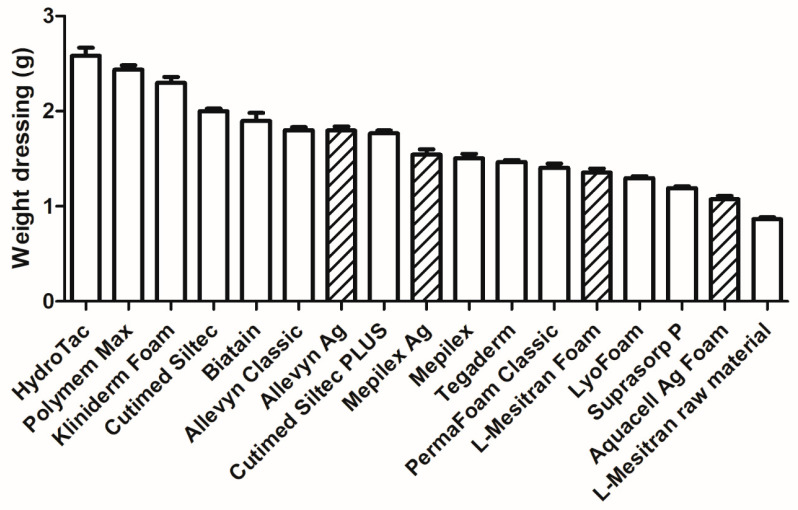
Weight of the different foam dressings (5 by 5 cm) in g. Twenty individual dressings were measured per product, and mean +/− SD is presented. The columns with diagonal lines are “active” foam dressings.

The weight of the dressings (25 cm^2^) varies between 2.582 g (Hydrotac) and 0.865 g (raw material of L-Mesitran Foam) (Figure 3). There is a big difference between dressing types, and there seems to be a correlation with the thickness and weight of the dressings. Aquacell Ag Foam, L-Mesitran Foam, and the raw material of L-Mesitran Foam are relatively thin and light.

### 2.4. Absorption Capacity

**Figure 4:**
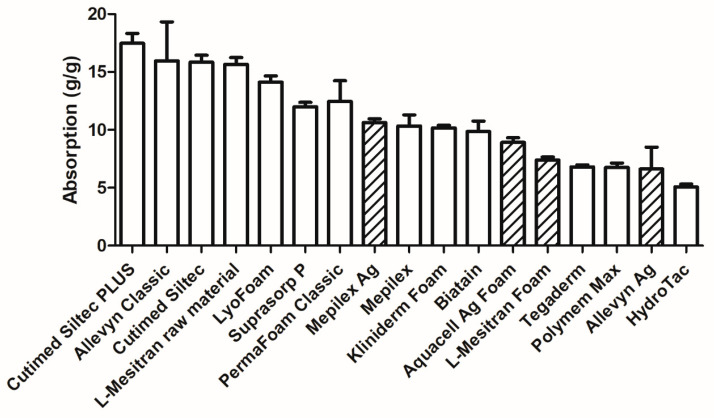
Absorption capacity of different foam dressings in g/g. Twenty individual dressings were measured per brand, and the mean +/− SD is presented. The columns with diagonal lines are “active” foam dressings.

The absorption capacity of all included dressings was assessed, see Figure 4. There is quite a difference between the different foam dressings, ranging from their lowest capacity of 5.1-fold absorption (g volume per g of dressing) for Hydrotac to 17.5-fold for Cutimed Siltec Plus. Four products are absorbing more than 15-fold their weight (Cutimed Siltec Plus, Allevyn Classic, Cutimed Siltec, and raw material of L-Mesitran Foam), and four products absorbing less than 7-fold their weight (Hydrotac, Allevyn Ag, Polymem Max, and Tegaderm). Interestingly, the active dressings are mostly at the lower end of absorption capacity (positions 8, 12, 13, and 16, out of 17). The question may arise whether the supplemented silver or honey to the foam dressings may be responsible for the relatively lower absorption capacity. Based on our measurements of the weights of the dressings (Figure 3), L-Mesitran Foam weighs 56% more than its raw foam dressing, which may explain why honey has a significant influence on the measured absorption capacity. This may not be the case for silver-based foam dressings because they contain a relatively low *w*/*w*% of silver. E.g., Aquacell Ag Foam contains 1.2% *w*/*w* ionic silver in the dressing [15], Mepilex Ag contains 1.20 mg silver/cm^2^ [16], and Allevyn Ag contains 1.99 mg/cm^2^ silver sulfadiazine [17]. In Figure 3, it can be observed that there is almost no difference in weight between the silver-loaded and its plain variant foam dressings (Mepilex vs. Mepilex Ag, and Allevyn Classic vs. Allevyn Ag, for Aquacell Ag Foam no plain variant was included). These weight differences between plain versus active (enriched) dressings may explain the differences between honey- and silver-based foam dressings but not the differences within the silver-based products. The absorption capacity of Mepilex and Mepilex Ag is similar (10.3 g/g and 10.6 g/g, respectively), while the absorption capacity of Allevyn Classic is 16.0 g/g and Allevyn Ag is 6.6 g/g (position 2 and 16, out of 17, respectively). There is no clear explanation for the large difference between the two silver-containing products from different brands. Potentially, it may be related to the silver formulation physically blocking the absorption into the pores/channels or it may be related to its physicochemical properties, e.g., its hydrophobicity.

### 2.5. Exudate Absorption Capacity of Foam Dressings

There are several qualitative and quantitative tools to determine the level of exudate production; Shih et al. recently published a review on this topic [18]. Qualitative tools are often based on categorical or numerical scales, e.g., exudate level categorized into “dry, minimal, moderate, or heavy”, or numerical scales where the patients are asked to rate the level of exudate on a scale of 0–10. Quantitative tools enable more reliable comparisons and include measuring the layers of gauze a wound soaks through, the number of dressing changes, and the degree of saturation of a dressing. In some cases, the exact volume is determined in cubic cm of drainage collected in surgical drains or by calculating the weight difference in a dressing before and after treatment [18]. Unfortunately, many of the existing tools have not been validated in robust studies [18].

**Figure 5:**
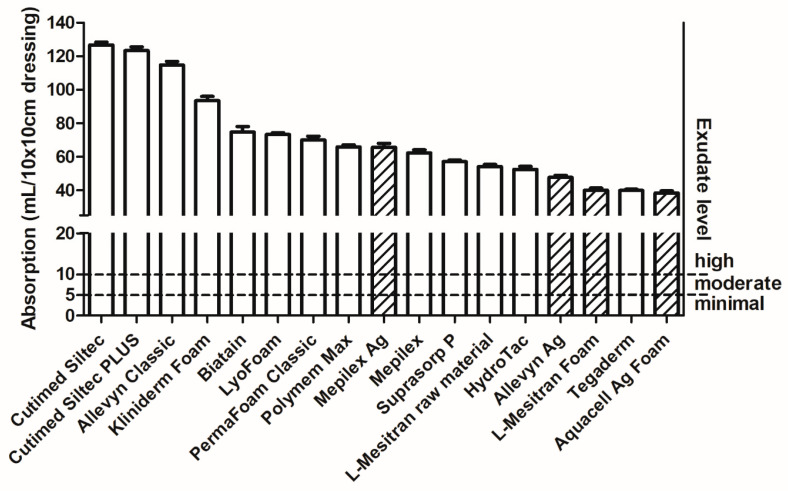
Absorption capacity per dressing. Data are presented in mL and corrected for 100 cm^2^ surface) in relation to minimal (<5 mL), moderate (5–10 mL), and high (>10 mL) levels of exudate. Twenty individual dressings of 5 × 5 cm (25 cm^2^) were measured per brand (and multiplied by a factor of 4 to obtain a 100 cm^2^ surface), with data are presented as mean +/− SD. The columns with diagonal lines are “active” foam dressings.

To assess whether the included foam dressings are capable of absorbing a certain level of exudate, we used a combination of categorical scoring with a quantitative volume, previously published by Mulder et al. [19], where the scores were: the amount of exudate as dry/absent, light/minimal (<5 mL per 24 h), moderate (5–10 mL per 24 h), and heavy/high (>10 mL per 24 h). The size of the wound or the dressing was not taken into consideration; however, a 10 cm by 10 cm dressing was used to visualize the saturation level. We can implement this into the measured absorption capacity of the included dressings and, hence, correct the g/g absorption capacity for 10 cm by 10 cm dressings (irrespective of their weight) and add visual lines of minimal, moderate, and high exudate levels. It is shown that all dressings can absorb high levels of exudate (Figure 5). The dressing with the lowest absorption capacity can still absorb 39.9 mL of exudate. When considering 10 mL or more per 24 h as ‘high levels’ of exudate, this dressing can absorb a higher volume.

Although the exudate level is only a rough estimation and the actual volume of exudate depends on many factors, e.g., evaporation, moisture vapor transmission (water vapor transmission rate), saturation of a dressing, gravity, and difficulties in precisely measuring the sizes and depth of a wound.

Other studies also use different ways to determine the amount of produced exudate, such as weighing the dressing before and after treatment, canister collection after negative pressure wound therapy, vapor pressure gradient (evaporative water loss), or by evaporimeter. Among these methods, the most relevant one seems to be measuring the dressings before and after treatment because this corresponds to the clinical situation where water vapor transmission and evaporation also occur. Using this method, Dealey et al. determined that leg ulcers produce 0.10–0.21 mL exudate per cm^2^ per 24 h [20]. When we use this information to calculate the exudate production for a 10 × 10 cm dressing, the volume would be 10–21 mL per 100 cm^2^ per 24 h. This is in line with the previously mentioned “high levels of exudate (>10 mL per 24 h)” and still far below the minimum absorption capacity of all foam dressings (39.9 mL for Aquacell Ag Foam).

### 2.6. Retention of Fluid

**Figure 6:**
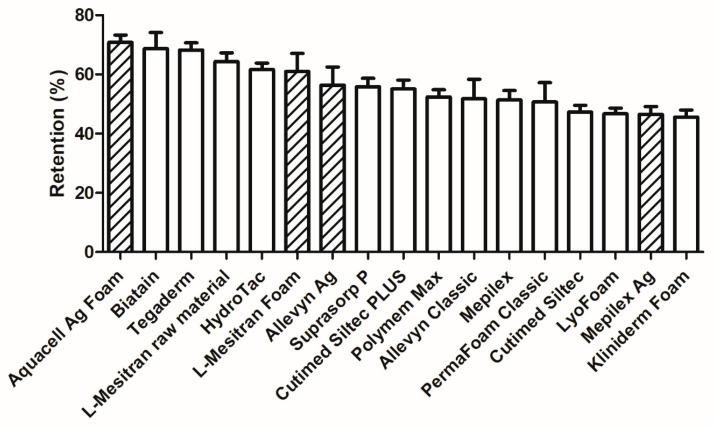
Retention of different foam dressings in %. Twenty individual dressings were measured per product, and the mean +/− SD is presented. The columns with diagonal lines are “active” foam dressings.

Another important aspect of foam dressings is their retention capacity, which helps prevent maceration and thus enhances healing. The retention capacity was measured for all dressings and varied between a minimum of 45.5% (Kliniderm Foam) and gradually increased to a maximum of 70.8% (Aquacell Ag Foam) (Figure 6). Interestingly, there is quite some variation among the different dressings and the distribution of the “active” foam dressings concerning their relative scores, with Mepilex Ag scoring the lowest at 46.5%, followed by Allevyn Ag at 56.4%, L-Mesitran Foam at 61.0%, and Aquacell Ag Foam at 70.8%. There is no significant difference or clear trend when comparing the active foam dressings to their plain versions: Allevyn Classic at 51.7% (−4.7% compared to the Ag version), Mepilex at 51.4% (+4.9% compared to the Ag version), and the raw material of L-Mesitran Foam at 64.3% (+3.3% compared to the medical-grade honey version).

### 2.7. Clinical Evaluation of L-Mesitran Foam by Wound Care Specialists

A group of eight registered nurses in the Czech Republic, all specialized in wound care, individually evaluated L-Mesitran Foam via a questionnaire. In total, 16 patients were treated with varying wound types: five leg ulcers (31%), four traumatic injuries (25%), three undefined chronic (hard-to-heal) wounds (19%), two burn wounds (12.5%), and two surgical wounds (12.5%). The state of the wounds varied, with 37% exhibiting yellow slough, 33% showing granulation tissue formation, and 12.5% reaching the epithelialization stage or being undefined. The median number of dressings per patient was 10 (range 6–20+), with a median treatment duration of 4 weeks (range 2 weeks– >6 months).

The participating clinicians rated the performance of L-Mesitran Foam. Exudate management was scored “very adequate” by 87.5% and “adequate” by 12.5% of the participants on the scale of “very inadequate, inadequate, neutral, adequate, and very adequate”. Retention was rated as very adequate for 75%, adequate for 12.5%, and no answer was given for 12.5%, using the same scale. The ease of dressing changes was rated as “very adequate” by 100% of the participants. There was no pain in 88% of the cases, while 12% experienced minor pain (score 4/5, with 5 being no pain). Other foam dressings commonly used by the participating wound care specialists consisted of Mepilex, Cutimed Siltec, Permafoam, and Aquacel Ag Foam. All participants (100%) were “very satisfied” when using L-Mesitran Foam compared to other foam dressings, and all of them (100%) confirmed that the layer impregnated with L-Mesitran Soft enhances the properties of the foam dressing. The participants’ impression of the product was positively impacted, and all stated they “very likely” will use the product again (Scale: very unlikely, unlikely, neutral, likely, and very likely).

Clinical observations reinforced these findings, with nurses noting improved wound healing and patient comfort (without pain or adverse events), particularly in previously stagnant wounds. The L-Mesitran foam demonstrated excellent exudate retention without causing maceration and was easy to remove (not incorporated into the wound bed). One specific case involved a patient with severe allergies and poor cooperation (low compliance), who showed significant improvement after using L-Mesitran Foam, experiencing reduced pain and increased willingness to comply with treatment.

### 2.8. Clinical Cases Supporting the Cost-Effectiveness of L-Mesitran Foam

The costs of treatment depend on many factors. Some factors are quantifiable, such as dressing costs and wound care time (time for direct local/topical care, wound cleaning, redressing, administration, etc.). However, there are also indirect costs, including losses to society and the quality of life for the patients. For example, if a treatment is ineffective, expensive surgery to amputate a limb, foot, or phalanges with a severely infected chronic wound may be necessary, or a patient may not be able to work due to a chronic wound or their mental state. These indirect costs are more difficult to quantify but will impact the total cost-effectiveness of a product. We selected several cases to present the wound healing-promoting activities of L-Mesitran Foam, as there is a causative relation between these properties and their cost-effectiveness. Faster and better healing wounds will decrease complications, healing time, wound care time, and automatic costs. All presented cases show that an excellent moist wound environment was created and maintained, supported by resolved maceration and a clear evolution of wound healing. Individual cases show that L-Mesitran Foam is effective in controlling exudate, debridement, resolving infections, and protecting the surrounding tissue against external stressors, and can be combined with compression and stay in situ for seven days. Together, this supports the cost-effectiveness of L-Mesitran Foam.

#### 2.8.1. Case to Support the Absorption/Retention Capacity Is Enough for Heavily Exuding Wounds

**Figure 7:**
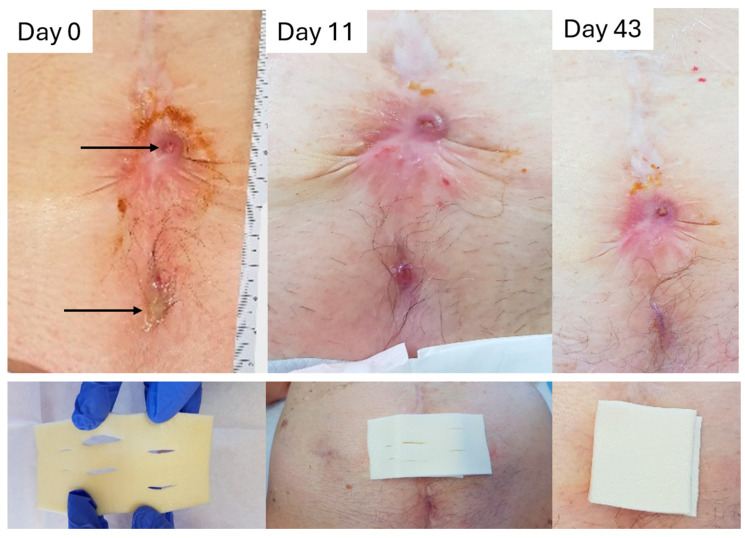
Wound progression of two eleven-month-old chronic abdominal wounds post-surgery in time. Treatment with L-Mesitran Foam was initiated and dressing changes were performed twice a week. In the first week, a double layer was applied, with the contact dressing being fenestrated; after one week, treatment was continued with a single layer. *The black arrows depicted in the “Day 0 photograph” indicate the two wounds. Pictures at the bottom illustrate the fenestration of the L-Mesitran Foam dressing and its application to the wound bed and covering with the second layer*.

An 81-year-old female presented with two chronic abdominal wounds following surgery that persisted for approximately eleven months (Figure 7). Previous treatments were provided in the hospital and by various health care practitioners, such as surgical debridement and colostomy bags to collect large amounts of exudate. Macroscopic examination of the wound indicated chronic inflammation, rolled wound edges, slough, and heavy exudate. Patient-related factors that could influence wound healing were being elderly, having renal and digestive problems, psychological issues (stress, sleep deprivation), and a partially dependent lifestyle. The top wound measured 1.0 cm in length and 1.0 cm in width, while the bottom wound measured 0.5 cm by 0.5 cm (Figure 7, see arrows). Signs of infection were non-healing, increased exudate levels, redness, and the presence of debris. A wound swab confirmed the scanty growth of *E. coli* and *Enterococcus faecalis*.

L-Mesitran Foam treatment was initiated, and dressing changes were performed twice a week. Due to the high amount of exudate and its high viscosity, two layers of L-Mesitran Foam were applied on top of each other, where the first layer in contact with the wound was fenestrated to allow lateral and vertical distribution of exudate in the first dressing and passage to the second dressing. After the second dressing change, the amount of exudate in the second layer was minimal, and treatment continued with only one layer of L-Mesitran Foam. After 11 days, the wound showed improvement with viable tissue, the absence of infection, low exudate levels, and maintained rolled edges. On day 43, the wound had reduced to 0.3 cm by 0.3 cm, and the wound edges improved. There were no signs of infection progression or increased exudate during treatment. This case shows that L-Mesitran Foam could absorb high levels of viscous exudate and retain it in the dressing when changed twice a week.

#### 2.8.2. Case to Support the Additional Benefit of Honey in Enhancing Debridement and Proliferation, and Why L-Mesitran Foam Is the First Choice to Combine with Compression Therapy

**Figure 8:**
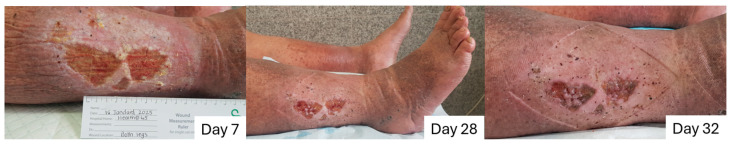
Progression of a chronic wound (lasting for >1 year) on the right leg over time. L-Mesitran Foam treatment was initiated and combined with modified compression therapy.

A 63-year-old male presented with spontaneous open wounds, located on the medial lateral aspect of the right leg (Figure 8, day 7). The wound had been present for over a year, and the patient treated himself with Silver Sulphadiazine 1% Cream and other over-the-counter ointments paid out of pocket (without reimbursement from public health insurance). The patient is diagnosed with varicose veins with ulcers and inflammation (CEAP classification 6S). Systemic conditions contributing to delayed healing were overweight (BMI of 29.8 kg/m^2^) and rheumatoid arthritis. The patient rates the pain level at 5 (on a visual analog scale of 0–10) during the day, increasing at night and varying between 6 and 8. Pedal pulses are present and assessed by Doppler. Locally, the wounds occupy a surface of 8.9 × 4.5, moderate, viscous exudate, rolled margins, sloughy, and low granulation, not odorous. There are typical clinical signs of infection, with inflammation, pain, and redness, and a wound swab confirmed infection with *Morganella morganii* (profuse growth), *Streptococcus agalactiae Group B* (moderate growth).

Treatment with L-Mesitran Foam commenced on the first visit, along with modified compression +/− 25 mmHg (due to high pain levels), twice a week. On day 28, the wound dimensions were reduced to 8.5 × 4.1 due to wound contraction and decreased inflammation. The wound bed improved with decreased levels of slough, and increased formation of granulation and epithelial tissue. The patient reported an absence of pain in the last 48 h, and the exudate consistency changed from a thick, viscous fluid to serous and reduced in quantity. The wound margins are still rolled, which is common for chronic wounds, and treatment continues with L-Mesitran Foam, and compression increased to +/−30 mmHg. On day 32, the patient reports no more pain, stating that his sleeping pattern has normalized, his mood has improved, and he feels more comfortable than he did over the past year. The wound area has reduced to 7.8 × 3.8, with the wound bed showing granulation, no slough, and present epithelial tissue. Exudate is low, the edges are advancing, and there are no clinical signs of inflammation or infection. Treatment continued with compression of +/−30 mmHg and L-Mesitran Foam.

#### 2.8.3. Case to Support the Additional Benefits of Honey in Resolving Infections and Protecting Against External Trauma

**Figure 9:**
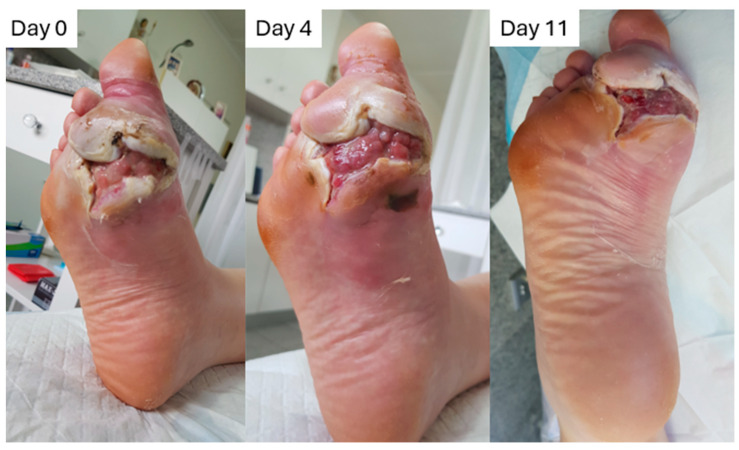
Progression of a diabetic foot ulcer in time. The wound was covered with L-Mesitran Tulle to ensure contact with the deeper laying wound bed and covered with L-Mesitran Foam for exudate control and protection against external forces/pressure. The patient was put on an offloading boot further relieving pressure and allowing wound healing.

A 42-year-old male with type 1 diabetes and a history of idiopathic abscess of right foot, which has been incised and drained in theater, presented to the wound clinic (Figure 9). Systemic factors influencing the healing process are dermatological, circulatory (peripheral vascular disease), metabolic, and impaired cognition. Doppler examination revealed the presence of dorsal, internal, and external malleolus pedal pulses on both feet. Influencing local factors include pain (score 7 on a scale from 0 to 10), deformity of the soft tissue due to the depth of the damaged structure, tunneling, and infection with two aggressive bacteria, i.e., Klebsiella *oxytoca* (moderate growth) and *Streptococcus dysgalactiae* (streptococcus group C, profuse growth). The wound presents viable tissue, with visible signs of infection (NERDS and STONEES), with no clinical suspicion of osteomyelitis. Wound size is 7.6 cm by 9.7 cm, with a measurable depth of 3.2 cm. This case has a few major challenges: (1) education of the patient, due to reduced cognition; (2) pressure offloading; (3) exudate control; (4) infection reduction; (5) protect newly formed tissue; and (6) anastomosis of the wound edges.

Due to the aggressiveness of the pathogens and the high risk posed by diabetic foot syndrome, systemic antibiotics (Augmentin, 1 g per 12 h for 10 days) were prescribed according to the sensitivity obtained from wound swabbing, and a strict protocol of local treatment commenced.

The objectives of wound management are, as aforementioned, to bring the wound edges together, to eradicate infection, to protect the new tissue, to control exudate, and to reduce pressure. To achieve this, the patient was put on an offloading boot, filled the spaces with L-Mesitran Tulle, brought the edges together with a wound closure system, and protected the wound area with L-Mesitran Foam dressing. The limb was further protected with a cotton wool bandage and retained with a semi-elasticated bandage. The reduction in the wound was remarkable, with dimensions of 5.5 by 8.5 and a depth of 2.1 cm on day 4. The exudate level decreased, with no staining of the exterior bandage, and the patient experienced a decreased pain level (score 3/10). On day 11, the wound size had further reduced to 3.6 cm by 6.4 cm, and healing was advanced in the absence of pain.

#### 2.8.4. Case to Support the Dressing Can Stay in Place for 7 Days

**Figure 10:**
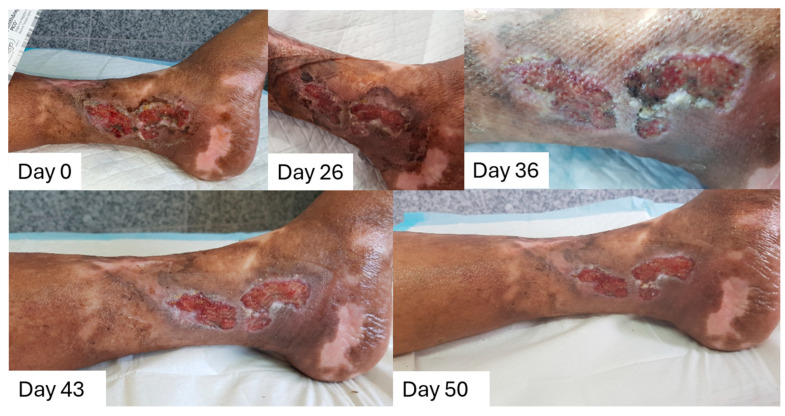
Wound progression of a chronic ulcer (>2 years) on the left lower limb over time. Treatment with L-Mesitran Foam was initiated, and dressing changes were performed twice a week. On day 26, debridement was carried out, and dressing changes were extended to every five days. Subsequently, on day 36, they were further extended to weekly changes.

A 68-year-old female with Type 2 Diabetes Mellitus, obesity (BMI of 31), and psychological conditions (depression, stress, anxiety, sleep deprivation) presented with a non-pressure chronic ulcer of the left lower limb. The wound had been present for approximately two years and its cause is unknown. Previous treatments included surgical debridement and negative pressure wound therapy at a different wound clinic. Local examination indicated the presence of chronic inflammation and advancing wound edges. The wound dimensions were 10.3 cm in length and 3.9 cm in width (Figure 10). The tissue is viable with the presence of debris, and the wound edges are advancing with no signs of deep compartment infection and low levels of exudate. The Doppler examination confirmed the presence of pedal pulses.

L-Mesitran Foam treatment was initiated with twice-weekly dressing changes. On day 26, wound dimensions were 9.8 cm by 3.7 cm and a clear healing progression was visible with debris and necrotic tissue effectively debrided. Due to the control of exudate, the dressing changes were extended to every 5 days (for 10 days), and since the exudate did not strike through the dressing, it was further extended to weekly changes (from day 36 onwards). This also accommodated the socioeconomic and mental challenges of the patient. On day 43, the wound size was slightly reduced to 8.3 cm by 3.5 cm, with tissue appearing cleaner and more viable, with low exudate levels and advancing edges. On day 50, the wound size had further decreased to 7.9 cm by 3.0 cm and continued to show viable tissue, low exudate levels, and advancing edges.

The use of L-Mesitran Foam dressing, with its ability to remain in situ for seven days, contributed to consistent wound size reduction and improved healing outcomes without signs of infection or increased exudate. This approach reduced the need for frequent dressing changes, enhancing patient comfort and minimizing clinical intervention frequency.

## 3. Discussion

Wound exudate management plays a pivotal role in optimizing wound healing, preventing complications such as maceration and infection (increased risk of multiplication of microorganisms in the exudate as their culture medium), and ensuring patient comfort. Foam dressings, whether used as primary or secondary wound dressings, are widely appreciated for several reasons (Table 3), including their absorptive capacity and ability to maintain a moist wound environment. Many different foam dressings exist, ranging from plain dressings to more sophisticated active foam dressings (enriched with other ingredients executing additional properties), with all major wound care companies producing these products (e.g., 3M, Paul Hartman, Smith & Nephew, and Essity). Our survey of wound care specialists indicates that health care professionals (physicians and nurses) utilize a variety of foam dressings, primarily from well-known brands. The extensive range of dressings available and the fact that most specialists employ multiple foam dressings suggest a lack of clear preference among them. Foam dressings are acknowledged for their effectiveness in managing exudate. Key physical properties of foam dressings in this respect to be considered are absorption and retention of exudate, creation of a moist wound environment, and the prevention of maceration. Other important physical properties that clinicians might consider are the thickness of the dressing, its capability to form a protective layer against potential external trauma, its role in preventing iatrogenic injuries, and its compatibility with additional therapies (e.g., active ingredients or compression therapy). Factors that can further enhance wound healing, although not typically associated with foam dressings themselves, include the stimulation of autolytic debridement, antimicrobial and prophylactic actions, and the promotion of wound healing. Moreover, a specific treatment preferably needs to be cost-effective and comfortable for patients. This study evaluated the physical properties of 17 commonly used foam dressings while also considering clinical parameters such as dressing change frequency, comfort, and cost-effectiveness.

During our survey, absorption was considered the most important characteristic of a foam dressing (79.5%), while retention (38.5%) and the frequency of dressing changes (41%) were viewed as secondary in importance. The absorption capacity of all tested foam dressings was sufficient for the hypothetical amounts of wound fluid across low, moderate, and high levels of exudate. Even the foam dressing with the lowest absorption capacity (39.9 mL per 24 h) could absorb almost four times the minimum amount of wound fluid classified as high-level exudate (10 mL per 24 h). The retention rate varied from 45.5% to 70.8%, indicating a difference of 25% between the best- and worst-performing dressings. It is difficult to determine a specific minimum required level of retention, as this also relates to the absorption capacity and the clinical situation. For instance, when subjected to mechanical stress during compression therapy, it could lead to leakage, resulting in maceration and secondary infections [9,21]. While there is no clear relationship between absorption and retention capacities, it is an interesting observation from our study that several dressings with the lowest absorption capacities (Aquacell Ag Foam, Tegaderm, Hydrotac) exhibit the highest retention capacities. Generally, the higher the absorption and retention values, the better the dressing performance. Dressings with lower retention capacity may require more frequent changes, increasing patient burden and treatment costs, potentially disrupting the wound healing process, e.g., by cooling of the wound, risking microtraumatization of the wound bed, and risking contamination [22,23]. The thickness of different dressings varies (3.2–6.0 mm); however, most products have a thickness from 5 to 6 mm, with a few exceptions, such as L-Mesitran Foam and Aquacel Ag Foam—those are thinner. The advantage of a thinner dressing is that, when used in combination with compression therapy, it will not leave indentations after application. Indentations increase the risk of developing new skin breakage, particularly in the vulnerable peri-wound area specific to chronic wounds.

### 3.1. Clinical Relevance—Strengths and Limitations

Despite the laboratory experiments being performed in a controlled setting, clinicians may consider the results of limited clinical relevance and have trouble selecting the most appropriate (suitable) foam dressings. For example, the absorption of real exudate may be different than the solution used in our experiments (an artificial saline solution that has a different viscosity and does not include contaminants), and the absorption angle and pressure in a Petri dish are different than on a moving patient, where bandaging, compression, or gravity may also play a role [24,25]. However, the strength of this study is that all foam dressings were compared under the same conditions. This should at least guide the clinician in selecting the dressing based on specific foam dressing characteristics. Of course, the clinician’s individual preference and experience with the products, their price, availability, and patient- and wound-specific factors may further influence the eventual dressing choice.

For infected wounds, an active dressing that has antimicrobial molecules incorporated in the dressing, such as silver- and honey-containing dressings, would be recommended. Similarly, when there is a risk of infection, using a dressing with known prophylactic activity would be advised. Active dressings can have additional benefits [14]. For example, medical-grade honey-based foam dressings release honey when in contact with the exudate. This will subsequently enter the wound and actively contribute to wound healing. We showed several cases in which chronic wounds, “stuck” in the inflammatory phase for months or even years, were moved to a healing path using active foam dressings. An overview of the cellular and molecular mechanisms regarding the different pro-healing characteristics of medical-grade honey has been published before [26,27]. Medical-grade honey differs from regular honey and follows strict criteria to guarantee its quality, safety, and efficacy [28], e.g., it is organic, free of contaminants, and gamma-irradiated. L-Mesitran Foam is a sterile, CE-marked, and FDA-approved product, and like other foam dressings, the shelf life is three years (Table 2).

### 3.2. Practical Considerations

The composition of wound exudate varies depending on the underlying physiological wound conditions, containing substances such as leukocytes, proteins (fibrin, fibrinogen, albumin, and globulin), electrolytes, glucose, inflammatory components, growth factors, enzymes (matrix metalloproteinases), wound debris, metabolic waste, and micro-organisms and their by-products. This influences its appearance and smell and may give valuable clues about the wound healing status, the presence of an infection, and how to advance healing. For example, fewer cells in the early stages result in clear, watery, pale-yellow exudate, indicating a low risk of infection. Bloody exudate is typically seen in fresh or healing wounds, indicating active inflammation or ongoing healing, as blood vessels remain open. Yellow or cloudy exudate suggests moderate infection with elevated levels of leukocytes and proteins. Green or brown exudate can indicate infection with bacteria such as *Pseudomonas aeruginosa*. Thick or sticky exudate is often caused by a higher level of debris, dead tissue, or microbial biofilms and is commonly present in non-healing or infected wounds [29].

Although foam dressings are intended for heavily exuding wounds, the viscosity of exudate can complicate its absorption as it can block the pores of the dressing. This is mostly experienced in chronic and infected wounds because the exudate is thicker and stickier [29]. An artificial saline solution was used to measure the absorption and retention capacity; however, this may not reflect the clinical situation of chronic wounds. Emphasizing that close monitoring of the dressing and wound conditions, including the exudate, is always important. To ensure the free passage of exudate, the dressings can be fenestrated before application to the wound bed and can be combined with another foam layer or other secondary absorbent bandage. For chronic wounds with viscous exudate, the thickness of the foam dressing is therefore less relevant, and all included dressings tested here would comply. In this light, using an active foam dressing with antimicrobial or debriding activity would be preferred over a regular foam dressing, as this would address the underlying reasons for the non-healing nature of the wound, i.e., decreasing infection, removing biofilms, and modulating proteinase levels. These effects are demonstrated in the presented clinical cases.

In our experience, honey will be released from the foam dressing when it comes in contact with the wound bed and will orchestrate several beneficial activities. Honey’s osmotic activity may stimulate exudate release from the underlying tissue, being responsible for creating an outflow and cleaning the wound bed. Honey’s low pH and antimicrobial properties will decrease the bacterial load, and together with its debriding properties, it will resolve microbial contamination and biofilms. Furthermore, honey’s pro-healing activity will advance healing by promoting granulation tissue formation, angiogenesis, and re-epithelialization.

### 3.3. Influence of Dressing Thickness and Patient Comfort

Foam is a good alternative to gauze and appears to be preferable regarding pain reduction, patient satisfaction, and nursing time [30]. Foam dressing thickness varies between 3.2 mm and 6.0 mm, which may have implications for clinical use. Thinner dressings, such as L-Mesitran Foam and Aquacell Ag Foam, may be more compatible with compression therapy, as they reduce the risk of indentation marks that can form new wounds, particularly in fragile or elderly skin. This aligns with previous findings that suggest thinner foam dressings distribute pressure more evenly under bandages [14]. Moreover, thinner dressings may be preferable for anatomical areas requiring greater flexibility and movement, improving patient comfort and adherence to treatment plans. Comfort is a key consideration for both patients and clinicians, as supported by the findings from our survey, which show that comfort for patients (69.2%) and ease of use (application/removal) (41%) are important in the selection of a dressing. Dressings that are too rigid or cause discomfort during movement can decrease compliance, subsequently prolonging wound healing. Soft, non-adherent dressings prevent the incorporation of new granulation tissue into the dressing, reduce pain upon removal, improve patient comfort, and facilitate healing.

### 3.4. Cost-Effectiveness Considerations

It is often thought that wound dressings are a major cost item for chronic wound care; however, research shows that wound care time and hospitalization account for 80–85% of the total costs [31]. It is important to recognize that the choice of wound care treatment and material significantly influences the total costs. For example, plain gauze may be inexpensive, but when this leads to infection or stagnation of healing, the wound requires more material, treatment, and wound care time, making it more expensive [30]. Three important cost drivers are healing time, frequency of dressing changes, and complications; using advanced materials that promote complication-free healing can constrain them [31]. The prices of foam dressings vary greatly, ranging from EUR 2.25 to EUR 8.96 per dressing. Active dressings, particularly those containing antimicrobial agents enriched with silver and honey, tended to be more expensive. However, the presented clinical cases support that honey-loaded foam dressings may offer additional cost-effectiveness due to their triple action: exudate management, antimicrobial properties, and promotion of wound healing. Multiple studies have highlighted the antimicrobial and healing benefits of medical-grade honey, particularly in chronic wound care, where biofilm formation is a concern [32,33,34,35]. Moreover, medical-grade honey is proven for its prophylactic activity [36,37,38]. Together, this will decrease the healing time and the required number of dressing changes and nursing time.

During our survey, 41% of the respondents considered the frequency of dressing changes important. The average frequency of dressing changes by these wound care specialists was daily (12.8%), every other day (23.1%), twice a week (43.6%), and once a week (20.5%). The “instructions for use” from most foam dressings stipulate that the dressings can stay in situ for 7 days; however, all include a side note that it depends on wound characteristics, and frequent monitoring is required to prevent maceration. The presented case reports illustrate that a 7-day wear time is feasible with L-Mesitran Foam. Furthermore, the frequency of dressing changes is correlated with the physical characteristics, such as absorption and retention capacity, influencing durability and cost-effectiveness. If a more expensive dressing offers prolonged wear time and fewer dressing changes, it may ultimately reduce overall treatment costs [30]. This finding is crucial for health care providers who need to balance clinical efficacy with budget constraints. For example, in a developing country, there is often a limited budget for health care, and reimbursement by insurance companies is less available.

Even among active dressings, there may be differences in activities. Previously, multiple clinical studies were performed to compare the antimicrobial activity of honey- and silver-based wound care products. A systematic review included nine randomized controlled trials that compared honey with silver sulfadiazine for the treatment of burns. They found that honey improved healing time by 5.76 days (95% CI: 3.39–8.14), the proportion of infected wounds rendered sterile (RR 2.59; 95% CI 1.58–2.88), and the level of evidence was of moderate quality. In an ex vivo human burn wound model, applying L-Mesitran Soft significantly improved re-epithelialization compared to silver sulfadiazine, while exerting similar antimicrobial effects against *P. aeruginosa* [39]. However, in a more recent study, there were no differences between L-Mesitran Soft and silver sulfadiazine in antimicrobial activity and reepithelialization, potentially attributed to changes in the formulation and slight differences in method, i.e., decreased treatment time from 3 weeks in the original study to 2 weeks in the latest study [40]. This may support why using silver-based products is usually advised for only two weeks [41]. Interestingly, in the same study, L-Mesitran Soft had a stronger antimicrobial activity than silver nitrate and a manuka honey-based product, while reepithelialization with manuka honey was impeded [40].

### 3.5. Shortcomings and Future Directions

While this study provides valuable insights into the performance of various foam dressings, it is important to acknowledge some limitations. Laboratory-based absorption and retention tests may not fully replicate real-life wound conditions, where factors such as patient movement, dressing occlusion, and wound exudate composition can affect performance. Nevertheless, the strength of this study lies in its standardized testing methodology, which enables direct comparison across multiple products.

Clinicians should consider both laboratory results and patient-specific factors when selecting foam dressings. Selecting a foam dressing with high absorption and retention capacity is critical for wounds with high exudate levels. Additionally, wounds at risk of infection may benefit from “active” dressings with additional antimicrobial ingredients, even if they are more expensive, due to their potential to reduce the need for further treatments. In this context, “active” foam dressings may offer additional benefits.

Furthermore, patient comfort, ease of use, and wear time must be considered. A dressing that provides high absorption but is difficult to apply or remove may not be favored by clinicians, particularly in patients with fragile skin (elderly or pediatric) [22]. Clinician feedback from this study highlighted absorption (79.5%), comfort (69.2%), retention (38.5%), and ease of use (41%) as the most critical factors in selecting foam dressings, underscoring the necessity of a balanced approach to product selection.

The clinical evaluation of L-Mesitran Foam highlights its strong clinical performance, particularly in managing exudate, reducing pain, and ensuring ease of use. The honey component is regarded as a valuable addition that further enhances wound healing outcomes. The overwhelmingly positive feedback indicates that L-Mesitran Foam could be an effective and preferred choice for wound care professionals. The representative clinical examples presented confirm these key properties of L-Mesitran Foam.

## 4. Materials and Methods

### 4.1. Questionnaire for Wound Care Specialists

During a major international wound care conference organized by the European Wound Management Association in London, UK, in 2024, 39 participants completed a short multiple-choice survey at the exhibition. In this questionnaire, the work position of the participant (e.g., physician, nurse, industry, other), the foam dressings they are familiar with (list of 20 different dressings with an option to specify others), the frequency of foam dressing changes (daily, every other day, twice a week, once a week), and what they consider the most critical aspect of a foam dressing (absorption, retention, change frequency, ease of use (application/removal), or patient comfort) were asked.

In a follow-up survey, eight wound care professionals were approached to complete a questionnaire, specifically about a recently released medical-grade honey-based foam dressing (L-Mesitran Foam, THEO Manufacturing, Maastricht, the Netherlands). The questions focused on the qualifications of the wound care professional (education and clinical setting), the treatment of the wounds (type, presentation of the wound, treatment duration, and the number of dressings used), dressing capacities (exudate management, retention, ease of use, pain level during application), and satisfaction compared to other foam dressings (open question about commonly used foam dressings, satisfaction compared to other foams, thoughts on whether honey has an additive effect, and likelihood of using the product again). At the end of the survey, the opportunity was given to leave open comments.

### 4.2. Patients and Treatments

In a retrospective study, clinical cases were collected in which the wounds were treated with L-Mesitran Foam. We selected the cases based on showcasing the beneficial properties of the dressing. In all cases, L-Mesitran was used as monotherapy except in one case at risk of systemic infection receiving antibiotics. All patients signed informed consent to use their data, including pictures for publishing, studies, or teaching materials, as long as their identity remains anonymous. The World Medical Association’s Declaration of Helsinki principles were followed (as updated in 2024).

### 4.3. Materials

Based on the questionnaire filled in by wound care specialists, 16 commercial foam dressings were selected in total; see Table 2. Most of them (*n* = 12) can be considered “plain” foam dressings without enrichment with additional ingredients. By contrast, some (*n* = 4) were considered more advanced “active” foam dressings (Mepilex Ag, Allevyn Ag, Aquacell Ag Foam, and L-Mesitran Foam) containing silver or medical-grade honey. For L-Mesitran Foam, the raw foam material (*n* = 1) without impregnated honey formulation was also included (see Table 2).

### 4.4. Laboratory Measurements to Assess the Physical Properties of the Dressings

All physical properties of the selected foam dressings were assessed according to the methods described in the European Norm (EN) 13726-2023: Test Methods for Wound Dressings. These include aspects of absorption, moisture vapor transmission, waterproofness, and extensibility [42]. All foam dressings were placed in their original boxes in the laboratory and acclimatized for at least 24 h before the experiments. The dressings were cut into 5 cm by 5 cm pieces for measurements.

The thickness of the dressings was measured using an electronic digital micrometer (Dasqua IP67). Twenty pieces of each foam dressing (5 cm by 5 cm) were measured individually. The weight of the dressings was measured using a balance (Steinberg Systems, model SBS-LW-300A, calibrated April 2024). Absorption and retention capacity tests were carried out in accordance with EN 13726-2023 [42]. Twenty samples of each foam dressing, cut into 5 cm by 5 cm pieces (25 cm^2^), were used to measure the absorption capacity. The baseline weight (W1) and thickness (T) of each sample were recorded without any liners or pouches and before absorption. Foam dressings (5 cm by 5 cm) were individually placed into a Petri dish with the skin contact layer to the glass side, and 40 mL preheated artificial saline solution (NaCl and CaCl2 in demineralized water) was added indirectly (not directly on the foam dressing but to the side) and subsequently incubated at 37.0 ± 2 °C for 30 min. After 30 min, dressings were taken out of the Petri dish using tweezers on the corner of the dressing, and leaking was allowed for 30 s before the sample was weighed (W2). The absorption rate was calculated with the formula: Absorption (g/g) = (W2 − W1)/W2. Once the absorption rate was measured, each sample was pressed by a 5 kg weight for 20 s and then weighed again (W3). Then, the retention capacity was calculated with the formula: Retention capacity (%) = (W3 − W1)/(W2 − W1) × 100%.

### 4.5. Statistics

Data of all parameters are presented as mean value ± standard deviation. Descriptive qualitative data provide a general overview of the different foam dressings. There is a threshold for how much wound exudate is produced by wounds at a certain time point. The absorption capacity of each dressing, therefore, needs to adhere to a specific absorption limit rather than being statistically significantly different from other foam dressings. The surveys conducted among wound care professionals (those attending the scientific conference and those evaluating the medical-grade honey-based foam dressing) were qualitative studies and were only descriptive.

## 5. Conclusions

Foam dressings remain a cornerstone in wound care, with significant variations in absorption, retention, thickness, and cost. Our study highlights the importance of choosing dressings based on both exudate management properties and clinical context. The findings support the use of advanced foam dressings containing antimicrobial agents, such as honey or silver, for enhanced cost-effectiveness and infection prevention. A survey among clinicians and a variety of clinical cases underscores the additional benefits of medical-grade honey in a foam dressing, demonstrating L-Mesitran Foam: (1) has adequate absorption and retention capacity for minimal-to-high exuding wounds; (2) creates a moist wound environment; (3) stimulates autolytic debridement; (4) can resolve infections; (5) protects fresh granulation tissue and prevents infection and trauma; (6) ensures optimal patient comfort and offers the option to combine it with compression therapy; (7) accelerates wound healing and reduces nursing time (e.g., remains in place for 7 days); (8) is cost-effective, and; (9) prevents iatrogenic adverse events. Further clinical studies are warranted to validate these findings in real-world settings.

## Figures and Tables

**Table 1:** Characteristics of Foam Dressings.

Description	Absorptive, sponge-like polymer dressings (with or without adhesive borders)
Composition	Polyurethane and other components
Key Properties	1. Absorptive 2. Provide a moist wound environment 3. Promote autolytic debridement 4. Provide cushioning effect against external trauma and mechanical forces (shear, pressure, and friction) 5. Carries active ingredients or can be used in combination with topical agents
Uses	Primary or secondary dressings on wounds (flat or cavity) with minimal to high exudate, where a non-adherent surface is important.

**Table 2:** Overview of foam dressings included in the evaluation. *NA: not available*.

#	Product Name	Description	Manufacturer (Reference #)	Lot#	Manufacturing/ Expiration Date	Size in cm (Number of Pieces Per Box)	Price Per Box in Euro (Price Per Piece/and, i.e., Corrected Per 100 cm^2^)
**PLAIN FOAM DRESSINGS**							
1	Tegaderm	High-Performance Foam Non-Adhesive Dressing	3M (90601)	33W66H	2023-12-16/ 2026-12-15	10 × 10 (10 pcs)	46,06 (4.61)
2	HydroTac	Foam dressing impregnated with gel	Paul Hartmann AG (685832)	300112113	2023-03-21/ 2026-03-01	10 × 10 (10 pcs)	31.60 (3.16)
3	Allevyn Classic	Non-adhesive hydrocellular foam dressing	Smith and Nephew (66800022)	202351	2023-12-01/ 2026-12-01	10 × 10 (10 pcs)	35.64 (3.56)
4	PermaFoam Classic	Foam dressing	Paul Hartmann AG (882000)	23061555	2023-10-20/ 2026-10-19	10 × 10 (10 pcs)	32.74 (3.27)
5	Cutimed Siltec Plus	Soft tack silicone foam dressing with superabsorbers	Essity (73288-01)	40720744	2024-02-13/ 2027-01-12	10 × 10 (10 pcs)	56.29 (5.63)
6	Cutimed Siltec	Silicone Foam dressing with super-absorbers	Essity (73285-01)	33630744	2023-09-06/ 2026-08-12	10 × 10 (10 pcs)	49.05 (4.91)
7	Lyofoam Max	Absorbent foam dressing	Mölnlyke Health Care AB (603201)	07332551952433	NA/ 2025-01-28	10 × 10 (10 pcs)	22.50 (2.25)
8	Biatain	Non-adhesive foam dressing—for wounds with extra fragile skin	Coloplast (33412)	9553318	2024-01-05/ 2027-01-4	10 × 20 (5 pcs)	33.69 (6.74/3.37)
9	Polymem MAX	Non-Adhesive Dressing	Ferris Mfg. Corp. (5045)	04423E2	NA/ 2028-02	11 × 11 (10 pcs)	89.55 (8.96/7.40)
10	Kliniderm Foam	Foam dressing for the treatment of chronic and acute wounds	Klinion (4017 4811)	321251-009	2023-11-30/ 2026-11-30	10 × 10 (10 pcs)	31.40 (3.14)
11	Surprasorb P	PU Foam Dressing, non-adhesive	Lohmann and Rauscher (20407)	2403312216	2024-01-07/ 2026-12-31	10 × 10 (10 pcs)	31.60 (3.16)
12	Mepilex	Soft silicone foam dressing	Mölnlycke Health Care AB (294200)	24154587	NA/ 2027-03-28	10 × 20 (5 pcs)	33.10 (6.62/3.31)
13	Raw material of L-Mesitran Foam	Polyurethane foam dressing without impregnated honey formulation	NA	NA	NA	Diverse (10 cm width, 100 m long)	NA

**ACTIVE FOAM DRESSINGS (additional properties)**							
14	Mepilex Ag	Antimicrobial soft silicone foam dressing	Mölnlycke Health Care AB (287221)	23332417	NA/ 2026-07-28	10 × 21 (5pcs)	32.37 (6.47/3.08)
15	Allevyn Ag	Non-adhesive antimicrobial hydrocellular foam dressing with a minimum of 1.99 mg/cm^2^ silver sulfadiazine	Smith and Nephew (66800084)	202405	2024-01-01/ 2026-01-01	10 × 10 (10 pcs)	88.27 (8.83)
16	Aquacell Ag Foam	Non-adhesive Hydrofiber Foam Dressing with Silver	Convatec (420642)	3K00197	10-2023/ 2026-10-01	10 × 10 (10 pcs)	72.43 (7.24)
17	L-Mesitran Foam	Non-adhering foam dressing	Theo Manufacturing BV (712.10)	202305X	2023-05/ 2026-05	10 × 10 (10 pcs)	39.48 (3.95)

**Table 3:** Desired Properties of Foam Dressings.

Most Suitable Product	1. Controls exudate levels (absorption and retention) 2. Does not become incorporated into the new granulation tissue of the wound 3. Serves as a cushion to protect the affected area 4. Provides a barrier against bacteria 5. Can be used in the case of infection 6. Suitable for wounds with hypergranulation 7. May be used during compression therapy 8. Easy to apply and remove 9. Cost-effective

## Data Availability

The data that support the findings of this study are available from the corresponding author upon reasonable request. All data relevant to the study are included in the article.
